# Impaired Glymphatic System Actions in Obstructive Sleep Apnea Adults

**DOI:** 10.3389/fnins.2022.884234

**Published:** 2022-05-06

**Authors:** Bhaswati Roy, Alba Nunez, Ravi S. Aysola, Daniel W. Kang, Susana Vacas, Rajesh Kumar

**Affiliations:** ^1^Department of Anesthesiology and Perioperative Medicine, University of California, Los Angeles, Los Angeles, CA, United States; ^2^Department of Medicine, University of California, Los Angeles, Los Angeles, CA, United States; ^3^Department of Radiology, University of California, Los Angeles, Los Angeles, CA, United States; ^4^Department of Bioengineering, University of California, Los Angeles, Los Angeles, CA, United States; ^5^Brain Research Institute, University of California, Los Angeles, Los Angeles, CA, United States

**Keywords:** DTI-ALPS index, apnea-hypopnea index, oxygen desaturation, diffusion tensor imaging, Epworth sleepiness scale

## Abstract

**Study Objectives:**

Obstructive sleep apnea (OSA) is accompanied by sleep fragmentation and altered sleep architecture, which can potentially hinder the glymphatic system, increasing risks for Alzheimer’s disease (AD), but the status is unclear in OSA. Our aim was to investigate the glymphatic system in OSA subjects and examine the relationships between OSA disease severity, sleep symptoms, and glymphatic system indices in OSA using diffusion tensor imaging (DTI).

**Methods:**

We acquired DTI data from 59 OSA and 62 controls using a 3.0-Tesla MRI and examined OSA disease severity and sleep symptoms with the Pittsburgh Sleep Quality Index (PSQI) and Epworth Sleepiness Scale (ESS). Diffusivity maps in the *x*-axis (D_xx_), *y*-axis (D_yy_), and *z*-axis (D_zz_), as well as in *x-y* axis (D_xy_), *y-z* axis (D_yz_), and *x-z* axis (D_xz_) were calculated, diffusion values for the projection and association fibers extracted, and the DTI analyses along the perivascular space (DTI-ALPS index) were performed. The glymphatic system indices were compared between groups and correlated with disease severity and sleep symptoms in OSA subjects.

**Results:**

D_zz_ values, derived from projection fiber areas, D_yy_ and D_zz_ values from association fiber areas, as well as ALPS and D_yzmean_ values were significantly reduced in OSA over controls. Significant correlations emerged between disease severity, sleep symptoms, and D_xy_, D_xx_, and D_zz_ values in OSA subjects.

**Conclusion:**

OSA patients show abnormal glymphatic system function that may contribute to increased risks for AD. The findings suggest that the APLS method can be used to assess the glymphatic system in OSA patients.

## Introduction

Obstructive sleep apnea (OSA) is a highly prevalent and progressive sleep disorder, affecting over 29 million American adults. The condition is characterized by recurrent episodes of complete or partial collapse of the upper airway with continued effort to breathe during sleep, which result in breathing pauses, creating O_2_ desaturation and re-oxygen cycles, enhanced sympathetic activity and intra-thoracic pressure, and leading to sleep fragmentation and impaired sleep architecture. The abnormal sleep architecture and sleep fragmentation can potentially hinder the sleep-assisted highly polarized cerebrospinal fluid (CSF) and interstitial fluid (ISF) transport system, known as the glymphatic system, which is more active during the sleep and facilitates brain extracellular waste removal, including beta amyloids, resulting from neural and cellular energy consumption (Xie et al., 2013).

Multiple studies have shown that OSA is linked with increased Alzheimer’s disease (AD) markers (Daulatzai, 2015; Lutsey et al., 2018; Bubu et al., 2019; Kitamura et al., 2020), including the beta amyloid levels (Bu et al., 2015; Sharma et al., 2018). These levels increase with time in non-treated OSA patients, suggesting that untreated individuals with OSA or OSA subjects with non-complaint continuous positive airway (CPAP) treatment are at heightened risk for developing AD (Li et al., 2015; Cholerton et al., 2016; Liguori et al., 2017; Mullins et al., 2020). Beta amyloid deposition eventually leads to brain tissue changes, including gray matter atrophy and clinical impairment, which might initiate in pre-symptomatic OSA individuals long before the hallmark symptoms of AD manifest. Thus, an understanding of glymphatic system activity in newly diagnosed OSA subjects might provide strategies to reduce beta amyloid deposition and delay risks for AD in the condition.

Overnight polysomnography (PSG) is the gold standard method for OSA diagnosis, and based on PSG studies, OSA disease severity can be categorized into mild, moderate, and severe, based on apnea–hypopnea index (AHI). Oxygen desaturation, an immediate consequence of OSA, results in intermittent hypoxemia and is considered as another marker of OSA severity. Most sequelae of OSA, including risk of diabetes and hypertension, are linked with AHI, as well as with degrees and duration of O_2_ desaturation. Although AHI has been shown to correlate with CSF beta amyloid levels (Sharma et al., 2018), direct associations between AHI, oxygen desaturation, and magnetic resonance imaging (MRI)-based glymphatic function indices are lacking.

The glymphatic system activities have been studied using invasive method of intrathecal administration of contrast agents in animals and humans (Iliff et al., 2012; Ringstad et al., 2017). However, MRI-based diffusion tensor image analysis along the perivascular space (DTI-ALPS) can examine the glymphatic system function (Taoka et al., 2017; Yokota et al., 2019; Yang et al., 2020). These procedures have been used to investigate the pathological changes of the glymphatic system function in AD (Taoka et al., 2017), and may also serve as a useful biomarker for monitoring glymphatic system function in OSA patients. In addition, DTI derived indices, including D_xx_, D_yy_, and D_zz_, can provide additional information about diffusivity in the right-left (*x*-axis), anterior-posterior (*y*-axis), and inferior-superior (*z*-axis) directions, and D_xy_, D_yz_, and D_xz_ may represent correlations of random motion between x-y, y-z, and x-z directions and show interactions between transverse, vertical, and longitudinal fiber directions (Le Bihan, 2003), but such indices have never been studied on OSA subjects.

Although a recent pilot study showed altered glymphatic system in limited number of OSA patients (Lee et al., 2022), but without examination of interactions between transverse, vertical, and longitudinal fiber directions, as well as correlations of glymphatic system indices with sleep symptoms. Therefore, in the present study, our aim was to examine the glymphatic system activity in newly diagnosed, treatment-naïve OSA patients compared to healthy control subjects, assess relationships between OSA disease severity, sleep symptoms, and glymphatic system indices in OSA patients, as well as correlations between diffusivity components and disease severity and sleep symptoms in OSA subjects.

## Materials and Methods

### Subjects

This study included 59 OSA and 62 healthy control participants. The demographic, physical, and other clinical variables of OSA and control subjects are summarized in Table 1. All OSA subjects were newly diagnosed *via* overnight PSG with at-least moderate severity [apnea-hypopnea-index (AHI) ≥ 15 events/hour], treatment-naïve for breathing condition, and were recruited from the accredited Sleep Disorders Center at the University of California Los Angeles (UCLA). OSA subjects with a history of stroke, heart failure, diagnosed brain condition, metallic implants, or body weight more than 125 kg (scanner limitation) were excluded. OSA subjects were not taking any medications, which could impact the cardiovascular system or mood regulation, such as α-agonists, angiotensin-converting enzyme inhibitors, vasodilators, or serotonin reuptake inhibitors. Control subjects were healthy and recruited through advertisements at the UCLA hospital system and Los Angeles area. Healthy control subjects lacked a clinical history of cardiovascular disease, thyroid disease, stroke, respiratory deficits, hypertension, renal dysfunction, drug and alcohol abuse, neurological or psychiatric conditions, and any use of cardiac or psychotropic medications that may introduce brain injury. To determine the potential for sleep disordered breathing or sleep disturbances in control subjects, control subjects, as well as their sleep partners were interviewed, when available. Control subjects were referred for an overnight PSG, if such a condition was suspected. All OSA and control subjects provided informed written consent prior to the study, and the protocol was approved by the Institutional Review Board of UCLA.

**TABLE 1:** Demographics and clinical variables of OSA and control subjects.

Variables	OSA (mean ± SD) [*n* = 59]	Controls (mean ± SD) [*n* = 62]	*p* values
Age (years) Sex (Male:Female)	49.9 ± 10.0 35:24	50.1 ± 10.4 34:28	0.91 0.62
BMI (kg/m^2^)	31.8 ± 5.5	26.2 ± 3.5	<0.001
Heart rate (beats/min) Systolic BP (mmHg) Diastolic BP (mmHg) BP Average	72.5 ± 12.6 126.3 ± 17.0 79.5 ± 10.6 102.9 ± 12.8	72.2 ± 9.2 115.1 ± 11.9 73.7 ± 8.3 94.4 ± 9.3	0.86 <0.001 0.001 <0.000
PSQI ESS	8.5 ± 4.1 (*n* = 57) 8.5 ± 4.4 (*n* = 58)	4.5 ± 2.6 4.7 ± 3.3	<0.001 <0.001
AHI	35.4 ± 21.0	–	–
SaO_2_ nadir baseline SaO_2_ ΔSaO_2_	78.5 ± 9.5 94.6 ± 2.1 16.1 ± 9.1	– – –	– – –

*OSA, obstructive sleep apnea; SD, standard deviation; BMI, body-mass-index; BP, blood pressure; PSQI, Pittsburgh sleep quality index; ESS, Epworth sleepiness scale; AHI, apnea–hypopnea index; SaO_2_, oxygen saturation as measured by blood analysis.*

### Overnight Polysomnography (PSG)

All OSA subjects underwent overnight sleep studies as part of clinical diagnosis, consisting of at-least 7-h monitoring period of electroencephalogram (central and occipital), electromyogram, electrocardiogram, right and left extra-ocular eye movement, thoracic and abdominal wall movement, air flow, O_2_ saturation, end-tidal CO_2_ levels, snore volume, bilateral leg movement, and sleep position. All PSG data were digitized and evaluated by a board-certified sleep physician at the UCLA Medical Center. The ratio of the total number of apnea and hypopneas to the total sleep time in hours were calculated to obtain AHI scores. OSA subjects with AHI values between 5–14 events/hour, 15–30 events/hour, and >30 events/hour were categorized as mild, moderate, and severe OSA, respectively (Goyal and Johnson, 2017, American Academy of Sleep Medicine [AASM], 2020). Lowest oxygen saturation levels, SaO_2_ nadir, and baseline SaO_2_ levels were obtained from the sleep study, and ΔSaO_2_ values were calculated by subtracting SaO_2_ nadir from SaO_2_ baseline.

### Sleep Quality and Daytime Sleepiness

We used two self-administered questionnaires to investigate sleep quality and daytime sleepiness in OSA and control subjects. Sleep quality was assessed using the Pittsburgh Sleep Quality Index (PSQI) (Carpenter and Andrykowski, 1998), and daytime sleepiness was evaluated with the Epworth Sleepiness Scale (ESS) (Johns, 1992). Both questionnaires are commonly used instruments for sleep quality and daytime sleepiness evaluation. A score > 5 on PSQI and > 10 on ESS were considered abnormal.

### Magnetic Resonance Imaging

Brain imaging data were acquired using a 3.0-Tesla MRI scanner (Siemens, Magnetom Prisma Fit, Erlangen, Germany), while participants lay supine. Foam pads were placed on both sides of the head to minimize head movement. High-resolution T1-weighted images were acquired using a magnetization prepared rapid acquisition gradient-echo (MPRAGE) pulse sequence [repetition-time (TR) = 2200 ms; echo-time (TE) = 2.4 ms; inversion-time = 900 ms; flip-angle = 9° matrix-size = 320 × 320; field-of-view (FOV) = 230 × 230 mm; slice-thickness = 0.9 mm; number of slices = 192]. Proton-density (PD) and T2-weighted images were collected in the axial plane, using a dual-echo turbo spin-echo pulse sequence (TR = 10,000 ms; TE1, 2 = 12, 124 ms; flip-angle = 130° matrix-size = 256 × 256; FOV = 230 × 230 mm; slice-thickness = 3.5 mm). Diffusion tensor imaging data were acquired using a single-shot echo-planar imaging with twice-refocused spin-echo pulse sequence (TR = 12,200 ms; TE = 87 ms; flip-angle = 90°band-width = 1,345 Hz/pixel; matrix-size = 128 × 128; FOV = 230 × 230 mm; slice-thickness = 1.7 mm, *b* = 0 and 800 s/mm^2^, diffusion directions = 30). We used lower *b*-value, 800 s/mm^2^ instead of 1,000 s/mm^2^, for DTI data collection for lower susceptibility artifacts and better signal to noise ratio, especially in diffusion images. We used the parallel imaging technique, generalized autocalibrating partially parallel acquisition, with an acceleration factor of two in all MRI data acquisition.

### Brain Image Evaluation and Analyses

We visually examined high-resolution T1-, PD-, and T2-weighted images of all OSA and control subjects for any brain pathology, such as tumors, cysts, or major brain infarcts. DTI images were also examined for any potential head motion-related or other imaging artifacts. None of the OSA and control subjects included in this study showed any such serious brain pathology or imaging artifacts.

We used the statistical parametric mapping package (SPM12^1^), DTI-Studio (v3.0.3) (Jiang et al., 2006), MRIcroN (Rorden et al., 2007), and MATLAB-based (MathWorks^®^) custom software for data processing and analyses.

### Diffusion Tensor Imaging Indices Calculation

We calculated the average background noise level from outside the brain parenchyma, using non-diffusion and diffusion-weighted images, and this noise threshold was used in all subjects to suppress non-brain regions during DTI indices calculations. We used diffusion (*b* = 800 s/mm^2^)-weighted images, collected from 30 diffusion directions, and non-diffusion (*b* = 0 s/mm^2^) images to compute diffusion tensor matrices using DTI-Studio software (Jiang et al., 2006). Diffusivity maps in the direction of the *x*-axis (D_xx_), *y*-axis (D_yy_), and *z*-axis (D_zz_) were calculated, in addition to D_xy_, D_yz_, and D_xz_ maps.

### Measurement of Analysis Along the Perivascular Space Index

**FIGURE 1:**
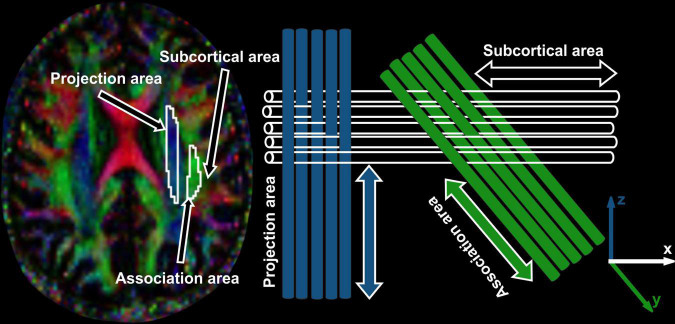
DTI color map with ROIs placed and arrows marked are showing projection, association and subcortical areas. Schematic diagram is showing the direction of the projection fibers (blue; *z*-axis), association fibers (green; *y*-axis), and the subcortical fibers (white; *x*-axis).

To evaluate DTI-ALPS index, we measured water molecules motion in the perivascular space direction by quantifying diffusivity along the direction of the perivascular space over those of projection and association fibers on an axial slice at the level of the lateral ventricles (Figure 1). At that level, the medullary veins run perpendicular to the ventricular wall, and the perivascular space runs in the same direction as the medullary veins, i.e., *x*-axis. On the axial level, projection fibers run adjacent to the lateral ventricle, and superior longitudinal fascicles, representing association fibers, run in the anterior-posterior direction outside the projection fibers. The direction of the perivascular space is perpendicular to the direction of both the projection fibers (*z*-axis) and the association fibers (*y*-axis) as shown in Figure 1. Thus, the diffusivity along the *x*-axis at regions with projection/association fibers will be predominantly represent the diffusivity along the perivascular space.

**FIGURE 2:**
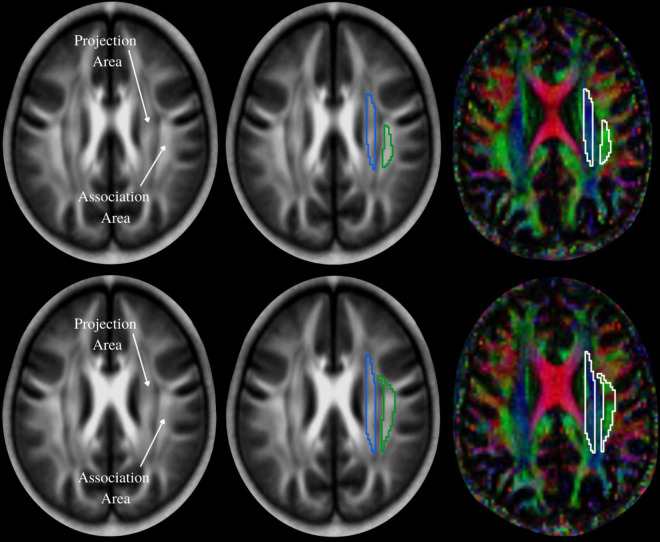
The periventricular white matter is divided into two regions; medial blue and lateral green areas, which correspond to the projection and association fibers, respectively. Region of interest (ROI) settings are placed on the projection and association fiber areas on FA and color-coded FA maps in two different slices. Values of co-registered maps were retrieved using these ROIs.

The diffusivity maps (D_xx_, D_yy_, D_zz_, D_xy_, D_yz_, and D_xz_) were normalized to Montreal Neurological Institute (MNI) space. Non-diffusion weighted (b0) images were normalized to MNI space using a unified segmentation approach, and the resulting normalization parameters were applied to the diffusivity maps. Two set of regions of interest (ROI) were placed at the level of the lateral ventricle body, in the area of the projection and association fibers (Figure 2) on the normalized diffusivity maps. The ROIs provided the value of D_xx_, D_yy_, D_zz_, D_xy_, D_yz_, and D_xz_ at projection and association fibers for each subject. Since all OSA and control subjects were right-handed, we obtained measurements only from the left hemisphere, as superior longitudinal fascicles and corona radiata fibers are thicker on the dominant side.

We calculated ALPS index, which is an indicator of the glymphatic system activity, from each OSA and control subject. Considering D_xx_ and D_yy_ in the area of projection fibers as D_xxpro_ and D_yypro_, respectively, and D_xx_ and D_zz_ at association fibers as D_xxasc_ and D_zzasc_, respectively, ALPS index was defined as: $ALPSindex=\frac{\left(D_{xxpro}+D_{xxasc}\right)/2}{\left(D_{yypro}+D_{zzasc}\right)/2}$, where (D_xxpro_ + D_xxasc_) /2 is expressed as D_xmean_ and (D_yypro_ + D_zzasc_) /2 as D_yzmean_.

### Statistical Analysis

Demographic and clinical data were assessed between OSA and control subjects by independent samples *t*-tests, and Chi-square test for categorical characteristics using the statistical package for the social sciences (SPSS, v 27.0, New York, NY, United States). All the diffusivity values and ALPS index were compared between OSA and control subjects using analysis of covariance (ANCOVA; SPSS software; covariates, age and sex) and corrected for multiple comparisons by using the Bonferroni correction method. Spearman’s correlations were used to determine associations, since some data were not normally distributed, between OSA disease severity, sleep symptoms, and glymphatic system indices within OSA subjects. A value of *p* < 0.05 was chosen to establish statistical significance.

## Results

### Demographic, Biophysical, and Clinical Variables

Demographics, biophysical, and clinical variables of OSA and control subjects are summarized in Table 1. No significant differences in age (*p* = 0.91) and sex (*p* = 0.62) appeared between groups. However, body mass index (*p* < 0.001) was significantly increased in OSA over control subjects. Also, the PSQI and ESS scores were significantly higher in OSA over controls (PSQI, *p* < 0.001; ESS, *p* < 0.001).

### Analysis Along the Perivascular Space and Diffusion Indices

**FIGURE 3:**
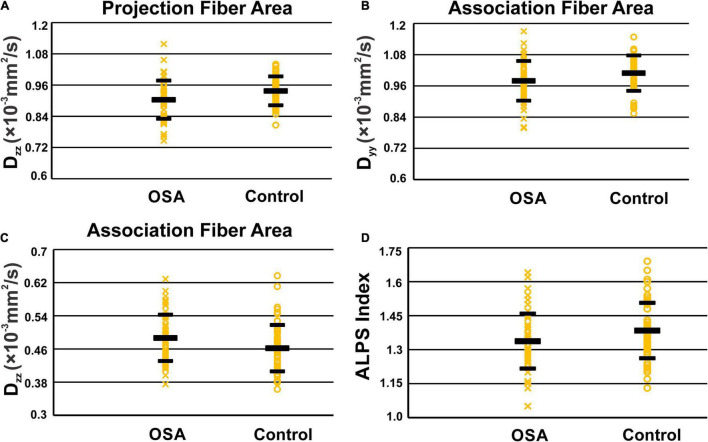
Scatter-plots of D_zz_ in periventricular projection fiber area **(A)**, D_yy_ **(B)**, and D_zz_ **(C)** in association fiber areas and ALPS indexes **(D)**. Only significant *P* values were shown in the figures.

Analysis along the perivascular space index was significantly decreased in OSA compared to control subjects (Figure 3 and Table 2). D_zz_ values, derived from projection fiber areas, were significantly reduced in OSA compared to control subjects (Figure 3 and Table 3). Diffusion changes in association fibers show significant reduction in D_yy_ and D_zz_ indices. Also, D_yzmean_ values were significantly different between OSA and control subjects (Figure 3 and Table 2).

**TABLE 2:** ALPS index, D_xmean_ and D_yzmean_ of the OSA and control groups corrected for age and sex with Bonferroni correction.

	OSA (mean ± SD)	Control (mean ± SD)	*P* value
ALPS median	1.34 ± 0.11	1.38 ± 0.11	0.03
D_xmean_ (× 10^–3^ mm^2^/s)	0.712 ± 0.04	0.708 ± 0.04	0.60
D_yzmean_ (× 10^–3^ mm^2^/s)	0.54 ± 0.05	0.51 ± 0.05	0.02

*ALPS, analysis along the perivascular space; D_xmean_, average of diffusivity along the x-axis in periventricular projection and association fiber area; D_yzmean_, average of diffusivity along the y-axis in periventricular projection and z-axis in association fiber area; OSA, obstructive sleep apnea; IQR, inter-quartile range; SD, standard deviation.*

**TABLE 3:** Diffusivity changes (corrected for age and sex with Bonferroni correction) in periventricular projection fiber area.

	OSA (mean ± SD) (× 10^–3^ mm^2^/s)	Control (mean ± SD) (× 10^–3^ mm^2^/s)	*P* value	OSA (mean ± SD) (× 10^–3^ mm^2^/s)	Control (mean ± SD) (× 10^–3^ mm^2^/s)	*P* value
	Periventricular projection fiber area			Periventricular association fiber area		
D_xx_	0.73 ± 0.04	0.72 ± 0.04	0.48	0.70 ± 0.07	0.69 ± 0.07	0.79
D_xy_	0.05 ± 0.01	0.04 ± 0.01	0.48	0.12 ± 0.03	0.13 ± 0.03	0.37
D_xz_	0.048 ± 0.02	0.046 ± 0.02	0.52	0.07 ± 0.02	0.06 ± 0.02	0.17
D_yy_	0.58 ± 0.05	0.57 ± 0.05	0.10	0.98 ± 0.07	1.01 ± 0.07	0.02
D_yz_	0.202 ± 0.02	0.20 ± 0.02	0.73	0.09 ± 0.03	0.10 ± 0.03	0.16
D_zz_	0.90 ± 0.06	0.94 ± 0.06	0.005	0.49 ± 0.05	0.46 ± 0.05	0.014

*OSA, obstructive sleep apnea; SD, standard deviation; IQR, inter-quartile range; D_xx_, diffusivity along the x-axis; D_xy_, diffusivity along the x-y axis; D_xz_, diffusivity along the x-z axis; D_yy_, diffusivity along the y-axis; D_yz_, diffusivity along the y-z axis; D_zz_, diffusivity along the z-axis.*

### Correlations Between Diffusion Indices, Sleepiness, and Obstructive Sleep Apnea Severity

**FIGURE 4:**
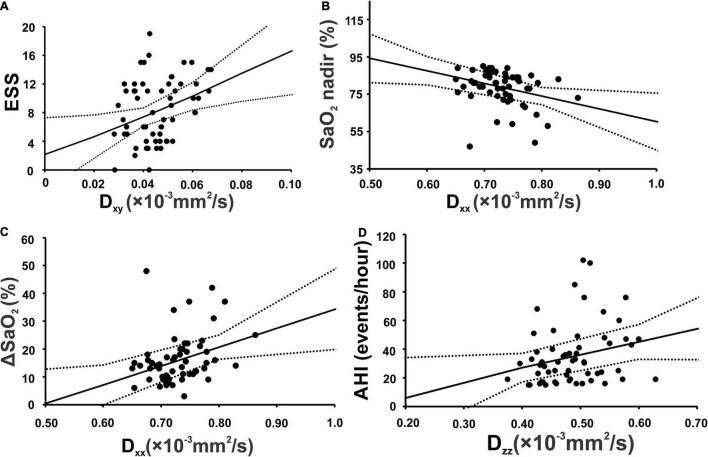
Scatterplots show significant correlation in projection fiber area between Epworth sleepiness scale and diffusivity along the *x-y* axis **(A)**, lowest oxygen saturation level of OSA patients (SaO_2_ nadir) and diffusivity along the *x*-axis **(B)** ΔSaO_2_ and diffusivity along the *x*-axis **(C)**. Along association fiber area, apnea-hypopnea index showed significant positive correlation with diffusivity along the *z*-axis **(D)**.

In periventricular projection fibers, D_xy_ values were significantly correlated with ESS scores, and D_xx_ values with SaO_2_ nadir and ΔSaO_2_ values in OSA subjects. At association fiber areas, D_zz_ was associated with AHI values in OSA patients (Figure 4 and Table 4).

**TABLE 4:** Correlation between periventricular projection and association diffusivity parameters and sleep severity and sleep symptoms in OSA subjects.

Region of interest	Variables	Correlation coefficient (*r*), *p*
Projection fiber area	ESS vs. D_xy_	0.29, 0.03
	SaO_2_ nadir vs. D_xx_	−0.34, 0.009
	ΔSaO_2_ vs. D_xx_	0.35, 0.006
Association fiber area	AHI vs. D_zz_	0.29, 0.03

*OSA, obstructive sleep apnea; ESS, Epworth sleepiness scale; D_xy_, diffusivity along the x-y axis; SaO_2_ nadir, lowest oxygen saturation level of OSA patients, D_xx_, diffusivity along the x-axis; ΔSaO_2_, difference between baseline and SaO_2_ nadir, AHI, apnea–hypopnea index; D_zz_, diffusivity along the z-axis.*

## Discussion

### Overview

Our study reports reduced diffusivity values, D_zz_ from projection fiber areas, and D_yy_ and D_zz_ indices from association fiber areas in OSA over control subjects. The ALPS index, an indicator of glymphatic system activity, was decreased, and D_yzmean_ values were significantly increased in OSA over control subjects. The daytime sleepiness scores were significantly associated with D_xy_ values and oxygen desaturation with D_xx_ values from periventricular projection fibers, and AHI with D_zz_ values from association fiber areas in OSA subjects, indicating role of sleep symptoms and disease severity in diffusion changes for the condition.

### Glymphatic System and Beta Amyloids

The glymphatic system is brain clearance system of metabolic wastes, including beta amyloids, resulting from metabolic neuronal activities (Murphy and LeVine, 2010; Jessen et al., 2015), and is dependent on fluid transport between the perivascular and interstitial spaces (Jessen et al., 2015; Liu et al., 2017). In the glymphatic system, CSF enters from subarachnoid spaces to brain parenchyma, along the perivascular space (PVS) (Jessen et al., 2015). The PVS around the arteries permits CSF to enter the interstitial spaces through aquaporin 4 (AQP4) regulated water channels, which are highly polarized to perivascular end feet and play a crucial role to maintain normal glymphatic function (Jessen et al., 2015; Benveniste et al., 2019). CSF entering the interstitial space rinses away waste proteins (Benveniste et al., 2019); CSF gets flushed out between cells flow into the PVS around veins and is discharged outside of the brain (Jessen et al., 2015; Benveniste et al., 2019).

Recently, “Central Nervous System (CNS) interstitial fluidopathy” concept has been suggested (Taoka and Naganawa, 2021) that signifies diseases in which abnormal interstitial fluid dynamics has major association with their pathology. Several disease processes have aspects of CNS interstitial fluidopathy, including Alzheimer’s and Parkinson’s diseases, traumatic brain injury, subarachnoid hemorrhage and ischemic stroke, and idiopathic normal pressure hydrocephalus. Based on the results from our study, it may be suggested that OSA patients has CNS interstitial fluidopathy characteristics.

### Sleep Disordered Breathing and Beta Amyloid Levels

Sleep and OSA issues are linked with reduced beta amyloid clearance (Osorio et al., 2011; Chen et al., 2018), though sleep problems and beta amyloid links are bidirectional (Roh et al., 2012; Spira et al., 2013; Ooms et al., 2014), which are suggested due to impaired glymphatic system (Iliff et al., 2012; Benveniste et al., 2019). Animal sleep studies show that beta amyloid is transported by the glymphatic system. Acute and chronic sleep issues contribute to beta amyloid accrual, and even one night sleep of deprivation in humans leads to higher amyloid burden in brain sites, including the hippocampus and thalamus (Roh et al., 2012; Prince and Abel, 2013; Liu et al., 2014; Ooms et al., 2014; Shokri-Kojori et al., 2018; Zhao et al., 2019).

Glymphatic system is influenced by aquaporin 4 (AQP4) gene (Iliff et al., 2012; Mestre et al., 2018), although role of AQP4 is still debated (Abbott et al., 2018; MacAulay, 2021). The criticism suggests that the flow resistance of the AQP4 channel is too large to enable bulk flow within the interstitial space, the arterial pulse in the tissue is too small to act as a driving force for bulk flow within the tissue, and high hydraulic resistance of the brain extracellular space would greatly restrict such flow. Also, there are other means by which to drain waste via the arterial wall that forms the intramural peri-arterial drainage pathway. However, the CSF and ISF have great importance in brain function and homeostasis.

During sleep, the interstitial space expands (∼60% of their wake-state diameter), allowing passing CSF through the brain parenchyma (Xie et al., 2013). High glymphatic system potentiation during sleep is dependent on the inhibition of noradrenergic projections from the locus coeruleus (Xie et al., 2013; Plog and Nedergaard, 2018); these pathways are suppressed during sleep, facilitating interstitial spaces to dilate and contributing to faster molecular motion across the brain parenchyma (Xie et al., 2013; Jessen et al., 2015). Several diseases, including type-2 diabetes mellitus, stroke, and sleep issues show that the glymphatic system is essential for maintaining brain homeostasis (Jiang et al., 2017; Rasmussen et al., 2018; Benveniste et al., 2019), and impaired function can contribute to beta amyloids deposition.

### Glymphatic System and Non-invasive Measures

Previous studies of the glymphatic system mostly have been performed on animals using two-photon laser scanning microscopy, which is an invasive procedure and suitable for a predetermined small perivascular space, but not suitable for whole-brain study, especially for deep brain tissues. Recent advancement in MRI techniques provide non-invasive whole-brain imaging of the glymphatic system. The initial study to monitor glymphatic function based on MRI was dynamic contrast-enhancement on rats, which demonstrated that MRI allows the identification of glymphatic pathways (Iliff et al., 2013). With introduction of new mathematical modeling, such as optimal mass transport, kinetic modeling using global input function and local input function, the MRI acquisition models were improved, and the first study on human to assess brain glymphatic function used intrathecal administration of gadobutrol as CSF tracer with MRI (Ringstad et al., 2017). With new MRI techniques based on diffusion imaging, the DTI-ALPS method has been developed, which is the method used in this study. Using this method, data can be acquired within several minutes, without injecting contrast agent; the procedure has the potential to monitor the glymphatic system status over time.

Diffusion tensor imaging-based analysis can examine glymphatic system activity by measuring diffusivity along the PVS (Taoka et al., 2017; Yokota et al., 2019). At the level of the lateral ventricles, the direction of the PVS is perpendicular to the ventricle wall, and is thus, mostly in the right-left direction (*x*-axis) on the axial plane. The PVS direction is also perpendicular to the direction of both the projection fibers (mostly in the *z*-axis) and the association fibers (mostly in the *y*-axis). Outside the superior longitudinal fascicles, subcortical fibers run mainly in the right-left direction in subcortical areas, and thus, the perivascular space runs perpendicular to the projection fibers and superior longitudinal fascicles. Since major fiber tracts do not run parallel to the direction of the PVS in this area, the arrangement of the perivascular space and major fibers allows independent analysis of the diffusivity along the direction of the PVS. In case of pathological changes along the right-left direction, both projection and associated fibers are equally affected (Taoka et al., 2017; Yokota et al., 2019). Thus, diffusion changes observed for both fiber bundles can be infer resultant from PVS pathology, and thus, from the glymphatic system activity (Taoka et al., 2017; Yokota et al., 2019). Using this newly developed method, significant links between water diffusivity along the PVS and cognition severity have been shown in AD (Taoka et al., 2017), but glymphatic system activity studies in newly diagnosed OSA subjects are lacking.

### Diffusion and Analysis Along the Perivascular Space Indices and Obstructive Sleep Apnea

In this study, the ALPS-index was calculated, which shows the influence of the water diffusion along the perivascular space and reflects activity of the glymphatic system. The results of our study were able to confirm with large number of subjects again with the findings of previous study (Lee et al., 2022) and show glymphatic system is dysfunctional in OSA subjects, with decreased ratio in OSA over healthy controls. Our results indicate that the ALPS-index is close to 1, which represents that the water diffusion along the perivascular space is minimal. The diffusivity components along the perivascular space direction in the projection fibers and association fibers are reduced in OSA, indicating overall altered perivascular diffusivity. The abnormal perivascular diffusivity might be due to altered sleep with multiple hypoxic episodes with acute stages (Kumar et al., 2014). In acute hypoxic stages, water diffusion parallel and perpendicular to the fibers decreases (Shereen et al., 2011), and results in reduced diffusivity components in perivascular space in the OSA condition.

### Correlations Between Diffusion Indices and Obstructive Sleep Apnea Symptoms and Severity

We observed significant positive correlations between D_xy_ and ESS scores and negative associations between SaO_2_ nadir and D_xx_ at projection fibers in OSA patients. Also, OSA patient showed positive correlations between AHI and D_zz_ at association fibers. These associations can be explained by altered white matter integrity that reflects the severity of myelin changes and systemic inflammation in OSA (Song et al., 2005; Chen et al., 2015). A previous study on AD observed similar associations between cognition and the diffusivity values along projection and association fibers (Taoka et al., 2017).

### Potential Pathological Processes

The possible pathophysiology of altered glymphatic system in OSA might involve systemic inflammation of various pathways, such as blood brain barrier dysfunction, increase in circulating cytokines, and oxidative stress by decreasing the convective flow, CSF-to-ISF turnover, resulting in impaired waste clearance. Astrocytes play a key role in the relationship between blood brain barrier and the glymphatic system, allow transport of nutrients between endothelial cells and the parenchyma by perivascular drainage through astrocytic water channel AQP4. The intermittent hypoxia and the invasion of peripheral inflammatory factors activate microglia and astrocytes, which secrete cytokines, such as IL-1β, IL-6, TNF-α, and HMGB1, oxidative species, adhesion molecules, and other signaling mediators and aggravate neuronal, axonal, and synaptic damage, increase myelin changes, and possibly compromise the AQP4 water channels in astrocytes and impeding the convective flow of the glymphatic system.

### Clinical Implications

These findings suggest the importance of early OSA diagnosis and treatment, since the disease can progressively change brain beta amyloids and subsequently induce AD neurodegeneration. OSA can be treated by clinical interventions that are currently available in clinical practice, such as continuous positive airway pressure (CPAP), which usually ameliorates OSA symptoms and improves long-term outcomes. Previous reports suggest that AD biomarkers are changeable, if treatment is initiated in the preclinical stage of the disease, when CSF beta amyloid metabolism and clearance are altered, but brain beta amyloid plaques have not yet been deposited (Liguori and Placidi, 2018).

### Limitations

One limitation of the study is that the healthy controls did not underwent for overnight PSG studies. The potential for sleep disordered breathing or sleep disturbances in healthy controls were assessed by interviewing control subjects and their sleep partners, as well as with sleep symptoms questionaries.

## Conclusion

Our study provides evidence of altered glymphatic system function in OSA patients based on ALPS index derived from DTI images. These abnormalities in glymphatic system may further contribute to increased risks for AD. These findings suggest the feasibility of using DTI-APLS method to assess glymphatic system activity in OSA patients.

## Data Availability Statement

The data underlying this article will be shared on reasonable request by the corresponding author.

## Ethics Statement

The studies involving human participants were reviewed and approved by the Institutional Review Board of UCLA. The patients/participants provided their written informed consent to participate in this study.

## Author Contributions

RK conceptualized the study plan and designed the study. BR collected and analyzed the data, interpreted the results, and wrote the manuscript. AN analyzed the data. RA, DK, and SV contributed in data collection, interpreting of the findings, and revising the manuscript. All authors read and approved the final manuscript for publication.

## Conflict of Interest

The authors declare that the research was conducted in the absence of any commercial or financial relationships that could be construed as a potential conflict of interest.

## Publisher’s Note

All claims expressed in this article are solely those of the authors and do not necessarily represent those of their affiliated organizations, or those of the publisher, the editors and the reviewers. Any product that may be evaluated in this article, or claim that may be made by its manufacturer, is not guaranteed or endorsed by the publisher.

## Footnotes

1
http://www.fil.ion.ucl.ac.uk/spm/
